# Predicting Disease Progression in Patients with Bicuspid Aortic Stenosis Using Mathematical Modeling

**DOI:** 10.3390/jcm8091302

**Published:** 2019-08-24

**Authors:** Darae Kim, Dongwoo Chae, Chi Young Shim, In-Jeong Cho, Geu-Ru Hong, Kyungsoo Park, Jong-Won Ha

**Affiliations:** 1 Division of Cardiology, Department of Medicine, Samsung Medical Center, Sungkyunkwan University School of Medicine, Seoul 03722, Korea; 2 Division of Pharmacometrics, Department of Pharmacology, Yonsei University College of Medicine, Seoul 03722, Korea; 3 Cardiology Division, Severance Cardiovascular Hospital, Yonsei University College of Medicine, Seoul 03722, Korea; 4 Cardiology Department, Ewha Womans University Medical Center, Seoul 03722, Korea

**Keywords:** bicuspid aortic valve, progression, mathematical model

## Abstract

We aimed to develop a mathematical model to predict the progression of aortic stenosis (AS) and aortic dilatation (AD) in bicuspid aortic valve patients. Bicuspid AS patients who underwent at least two serial echocardiograms from 2005 to 2017 were enrolled. Mathematical modeling was undertaken to assess (1) the non-linearity associated with the disease progression and (2) the importance of first visit echocardiogram in predicting the overall prognosis. Models were trained in 126 patients and validated in an additional cohort of 43 patients. AS was best described by a logistic function of time. Patients who showed an increase in mean pressure gradient (MPG) at their first visit relative to baseline (denoted as rapid progressors) showed a significantly faster disease progression overall. The core model parameter reflecting the rate of disease progression, α, was 0.012/month in the rapid progressors and 0.0032/month in the slow progressors (*p* < 0.0001). AD progression was best described by a simple linear function, with an increment rate of 0.019 mm/month. Validation of models in a separate prospective cohort yielded comparable R squared statistics for predicted outcomes. Our novel disease progression model for bicuspid AS significantly increased prediction power by including subsequent follow-up visit information rather than baseline information alone.

## 1. Introduction

Bicuspid aortic valve (BAV) is one of the most common congenital cardiac diseases [1,2]. Prior studies analyzed the risk of progression of aortic stenosis (AS) or aortic dilatation (AD) in BAV, based on baseline information, and demonstrated that future surgical intervention was positively correlated with age and moderate to severe valve dysfunction at baseline [1,3]. In patients with calcific AS, an association between hypercholesterolemia and AS progression has been reported, suggesting that progression of AS is similar to that of atherosclerosis [4,5], although prospective trials failed to prove protective role of statins against AS progression [6,7,8].

As of yet, no quantitative prediction models of the disease progression trajectories of AS and AD in BAV have been reported in the literature. Assessing the non-linearity of disease progression is necessary to predict the future disease trajectory from serial echocardiographic measurements. Considering the degenerative nature of AS, the function and morphology of the aortic valve (AV) would deteriorate rapidly over time with progression of AS. Therefore, including information acquired from subsequent visits as opposed to baseline factors alone in the analysis may be more appropriate.

In this study, we aimed to answer two specific questions: (1) Can future trajectories of the progression of AS and AD be linearly extrapolated from a few serial echocardiographic measurements? If not, what is an effective way to predict the non-linear progression of AS and AD in BAV patients? (2) Would incorporating the time-varying status of disease into the prediction model improve the accuracy of individual predictions? By answering these questions, we aim to propose a method to determine the expected time to reach criteria for intervention for severe AS and AD [3,9,10].

## 2. Materials and Methods

### 2.1. Study Population

After excluding patients with significant aortic regurgitation (AR) at baseline or prosthetic valves, 169 bicuspid AS patients with at least two serial echocardiograms were enrolled. Clinical data and laboratory data at the time of first echocardiogram were obtained. If patients underwent valve surgery or an interventional procedure during the follow-up period, echocardiographic data obtained just before the surgery was used to assess the progression of AS. Patients were randomly assigned into the training group (*n* = 126) or test group (*n* = 43) in a ratio of three to one. The AS disease progression model was derived from the training group of 126 patients, and the model was validated using the test group of 43 patients. This study was approved by the institutional review board of severance hospital, Seoul, Korea.

### 2.2. Echocardiography

All patients underwent two-dimensional transthoracic echocardiography. The first echocardiography when bicuspid AS was diagnosed was used as baseline. BAV was diagnosed when only two cusps were unequivocally identified on the short-axis view, with a systolic doming appearance on the parasternal view [3,11]. The type of BAV was determined based on the presence of raphe according to previous studies [11,12]. Type 1:1 raphe with a fusion of the left coronary and right coronary cusps; type 2:1 raphe with a fusion of the right coronary and noncoronary cusps; type 3:1 raphe with a fusion of the left coronary and noncoronary cusps; and type 0:no raphe with 2 developed cusps. AS assessment was performed according to current guidelines [13,14]. Two-dimensional ascending aorta measurements were recorded in systole at the sinus of Valsalva (SoV), the sinotubular junction (STJ), and the proximal ascending aorta (AAo) [15,16]. A follow-up echocardiography was performed according to clinicians’ decision according to patients’ symptoms and severity of disease. The median first visit time after baseline measurement was 12.4 months, with 5% and 95% percentiles of 3 weeks and 48.8 months, respectively.

### 2.3. Analysis Variables

Variables used for analysis were mean pressure gradient (MPG, mmHg) for AS progression and the aortic diameters (mm) of SoV, STJ, and AAo for AD progression. Time was expressed in months.

### 2.4. Aortic Stenosis (AS) Progression Model

#### 2.4.1. Basic Model

Linear and nonlinear models were fitted to serial MPG measurements from the training dataset. The following models were tested for MPG predictions:

MPG(t) = MPG_0_ + SL × t (1)

MPG(t) = MPG_0_ + (MPG_∞_ − MPG_0_) × (1 − exp(α × t)) (2)

(3) $$\mathrm{MPG}(t)\;=\;\frac{{\mathrm{MPG}}_{\infty }}{1+\left(\frac{{\mathrm{MPG}}_{\infty }}{{\mathrm{MPG}}_{0}}-1\right)\times \exp\left(-\alpha \times t\right)}$$

where

MPG_0_: baseline MPG (= MPG(0))
MPG_∞_: asymptotic maximum MPG (= MPG(∞))
SL (slope): rate of MPG increment (= ∆MPG/∆t)
α: rate constant of MPG increment.

**Figure 1:**
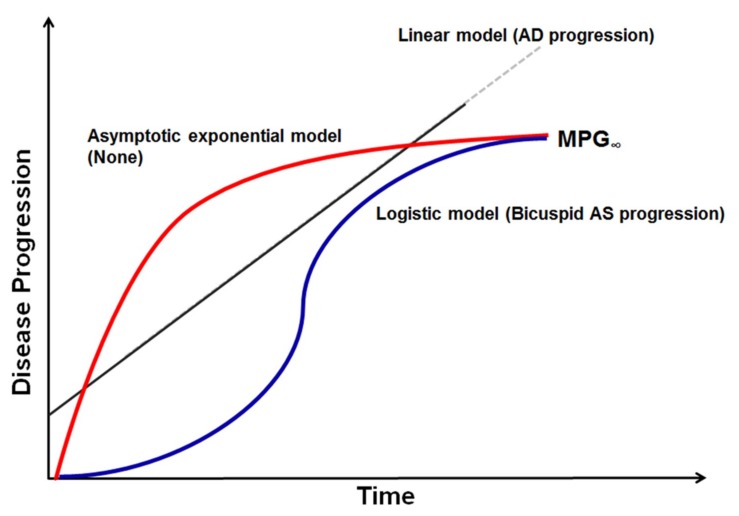
Graphical representation of disease progression model. Mean transaortic pressure gradient and aortic diameter measurements were used in disease progression modeling for bicuspid aortic stenosis (AS) and aortic dilatation (AD). Among linear, asymptotic exponential, and logistic models tested, logistic models best described bicuspid AS progression and a simple linear disease progression model best described AD progression. MPG—mean pressure gradient.

Equations (1)–(3) will be referred to as linear, asymptotic exponential, and logistic disease progression models, respectively (Figure 1).

The linear disease progression model assumes that MPG increases linearly with time. With this model, predicting the future time course of echocardiographic endpoints would be simple, since the treating physician could linearly extrapolate from past serial measurements to make future predictions. On the other hand, if there was significant non-linearity associated with the disease progression, more elaborate methods would be needed. Asymptotic exponential and logistic progression models assume that MPG approaches a certain value, MPG∞, with time. The main difference between the two is in the shape of the disease progression trajectory. The asymptotic exponential disease progression model assumes that the rate of MPG increment is greatest in the beginning and decreases thereafter. The logistic disease progression model assumes an S-shaped trajectory of MPG increment, where its rate is slow in the beginning, becomes faster with time, and then slows again as MPG approaches the upper bound. For MPG_0_ in Equations (1)–(3), the observed value was used, with assay error incorporated.

#### 2.4.2. Incorporation of Information from Subsequent Visits

After selecting the model from Equations (1)–(3) that best describes the data, we investigated whether subsequent follow-up visit information can be used to improve our predictions. To this end, an indicator of whether MPG increased from baseline to the first follow-up visit, denoted by λ, was defined as follows:

(4) $$\lambda =\mathrm{MPG}\prime \left(t_{1}\right)/{\mathrm{MPG}}_{0}=\;\frac{\mathrm{MPG}\left(t_{1}\right)-{\mathrm{MPG}}_{0}}{t_{1}\times {\mathrm{MPG}}_{0}}$$

In the above equation, t_1_ is the time of the first visit elapsed since the time of the baseline (t_0_), and $\mathrm{MPG}\prime \left(t_{1}\right)$ is the derivative of MPG(t) at t_1_.

Then, assuming that patients can have different values of SL or α (Equations (1)–(3)) depending on the degree of MPG increment, the following adjustment for predictions were made:

(5) $$\alpha \;=\;\{\begin{array}{l} \theta_{1}\;\;\;\;\;\mathrm{for}\;\lambda \leq e\\ \theta_{2}\;\;\;\;\;\mathrm{for}\;\lambda \;>e \end{array}$$

In the above, e is the optimal division boundary determined using a grid search algorithm. Values ranging from 0 to 0.5 with a step size of 0.05 were tested. $\theta_{1}$ and $\theta_{2}$ are SL or α estimated from the two subgroups. For example, taking Equation (2) as the basic disease progression model, the MPG trajectories of patients with λ ≤e would be described as $\frac{{\mathrm{MPG}}_{\infty }}{1+\left(\frac{{\mathrm{MPG}}_{\infty }}{{\mathrm{MPG}}_{0}}-1\right)\times \exp\left(-\theta_{1}\times t\right)}$ (see Equation (3)) while those with λ >e as $\frac{{\mathrm{MPG}}_{\infty }}{1+\left(\frac{{\mathrm{MPG}}_{\infty }}{{\mathrm{MPG}}_{0}}-1\right)\times \exp\left(-\theta_{2}\times t\right)}$. To acquire a good estimate of e, non-linear mixed effects modeling was undertaken so that random intra-patient variability could be separated from random inter-patient variability.

### 2.5. Aortic Dilatation (AD) Progression Model

As was done for MPG, all three disease progression models (linear, asymptotic exponential, and logistic disease progression models) were tested for SoV, STJ, and AAo or with model formulations similar to those defined in Equations (1)–(3).

### 2.6. Model Validation

As stated above, the training dataset consisting of 126 patients was used for model building, and the test dataset of the remaining 43 patients was used for model validation. The null model was set to a simple linear model with which other candidate models were compared. Four-fold cross-validation was performed on the training dataset to ensure that improvements in model fit using the more complex models (i.e., asymptotic exponential or logistic) were not due to overfitting. Model parameters were fixed at the values obtained from the training dataset, and adjusted R-squared values were used to determine how well the model predicted values in the test group. We have chosen R-squared as a performance metric because ideally, predictions and observations should lie on the line of unity with minimal scatter (i.e., linear regression of observations on predictions should result in $R^{2}$ = 1). Moreover, interconversion between R-squared and mean squared error is possible via the following formula:

(6) $$1\;-\;R^{2}\;=\;\frac{\sum {\left(observation-prediction\right)}^{2}/N}{{\left(observation-mean\right)}^{2}/N}\;=\;\frac{\mathrm{MSE}}{\mathrm{Var}\left(y\right)}$$

where MSE stands for mean squared error and Var(y) stands for variance of the observations.

### 2.7. Software

Data exploration, analysis, and simulation were carried out using NONMEM version 7.3 (ICON, https://iconplc.com), PsN version 4.2 (Uppsala university, Sweden, https://uupharmacometrics.github.io/PsN/), and R version 3.4.2 (https://www.r-project.org/). Categorical variables, expressed in percentages or frequencies, were compared using the χ^2^ test or Fisher’s exact test between rapid and slow progressors. Continuous variables, expressed in either mean ± standard deviation or median with range, were compared using Student’s t test between two groups.

## 3. Results

### 3.1. Patient Population

The mean observation period was 59.2 months (range, 12–150 months), and the final population consisted of 169 patients. There were no significant differences based on Student’s t test and χ^2^ test or Fisher’s exact test in the clinical and echocardiographic variables of the training group (*n* = 126) and validation group (*n* = 43), except higher estimated glomerular filtration rate, as described in Table S1. The median age was 61 years (27–86), and 74 patients in the training group (59%) were men. Patients in the training group had a mean MPG of 27.9 mmHg, and the mean values of SoV, STJ, and AAo were 34.5 mm, 29.4 mm, and 42.0 mm, respectively.

### 3.2. Disease Progression Model

When linear, asymptotic exponential, and logistic models were tested for the AS progression model, the logistic model resulted in the highest log-likelihood. Upon four-fold cross-validation, the difference of objective function value (OFV), defined as the −2 log likelihood of sums of extended least squares, between logistic and linear disease progression model in the validation datasets was 14.1, with an associated *p*-value of 0.00017 (see Table S2). Hence, logistic progression model was selected. Another piece of evidence favoring logistic over linear disease progression model comes from a two-stage analysis where simple linear regression of MPG on time was carried out on an individual basis (see Figure S1). The estimated slope coefficient was then stratified on baseline MPG and its mean values were examined (Table S4). The estimated slope increases from 0.17 mmHg/month, when baseline MPG is 5–10 mmHg, to 0.26 mmHg/month, when baseline MPG is 20–30 mmHg. In patients with baseline MPG 30–40, the slope decreases to 0.16 mmHg/month and becomes virtually 0 in patients with baseline MPG 40–50 mmHg. This suggests that the rate of AS progression is nonlinear and depends on the MPG value at the time of assessment.

For the AD progression model, neither the asymptotic exponential or the logistic disease progression model led to a significantly better fit than a simple linear model. Therefore, for SoV, STJ, and AAo, a simple linear disease progression model would suffice.

### 3.3. Time to Indication for Intervention 

#### 3.3.1. Time to AS Intervention

An MPG of 40 mmHg is considered an indication of severe AS [16]; and, from a therapeutic perspective, it would be useful to predict the time required for a patient to reach an MPG of 40 mmHg (T_40_). Accordingly, T_40_ was calculated from Equations (1)–(3). For example, T_40_ for the logistic disease progression model can be obtained from Equations (2) as follows:

(7) $$T_{40}=\log\left(\frac{40\times \left({\mathrm{MPG}}_{\infty }-{\mathrm{MPG}}_{0}\right)}{{\mathrm{MPG}}_{0}\times \left({\mathrm{MPG}}_{\infty }-40\right)}\right)/\alpha$$

#### 3.3.2. Time to AD Intervention

Similarly, an aortic diameter of 55 mm is considered a threshold for surgical intervention, [17] and T_55_ was predicted based on the selected model. For example, T_55_ for the linear disease progression model can be obtained from Equation (1) as follows:

T_55_ = (55 − D_0_)/SL (8)

D_0_: Diameter of SoV, STJ, or AAo at baseline.

### 3.4. AS Progression Model

When only baseline information was incorporated, the AS progression model yielded an α estimate of 0.0091/month in non-type 2 BAV phenotype (type 0, type 1, and type 3) and 0.0045/month in type 2 BAV (data not shown). No statistically significant difference of the α estimates was found between types 1 and 3, 1 and 4, or 2 and 4, based on post-hoc ANOVA and multiple Student’s t tests of the α estimates. The mean individual estimates of α in BAV types 1, 2, 3, and 4 were 0.012, 0.0056, 0.017, and 0.010/month, respectively.

The grid search returned the optimal value of e (see Equation (5)) as 0. The incorporation of first visit information led to a decrease in AIC (Akaike Information Criteria) that was highly significant (*p* < 0.001) compared to the model using baseline data alone. When using this model, there were two distinct patterns of AS progression that were classified as rapid and slow progressors, as shown in Table 1.

**Figure 2:**
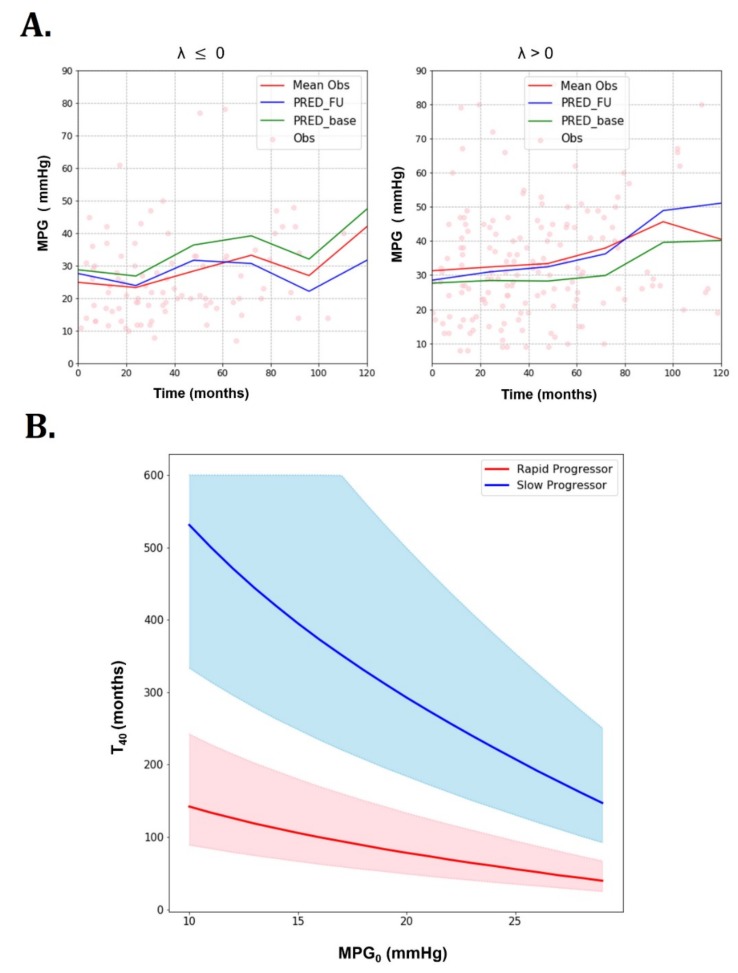
Goodness of fit plots of the AS progression model and $T_{40}$ predictions for rapid progressors (λ > 0/month) and slow progressors (λ ≤ 0/month) (training dataset). (**A**) Goodness of fit plots (dots: observation, red line: mean observation, blue line: mean prediction with incorporation of subsequent visit information, green line: mean prediction with only baseline information). (**B**) $T_{40}$ predictions for rapid progressors (λ > 0/month) and slow progressors (λ ≤ 0/month) (training dataset): Rapid and slow progressor show different $T_{40}$ predictions (thick lines: means, shaded areas: 50% prediction intervals).

Rapid and slow progressors were determined by the rate of MPG change (λ) from baseline to the first follow-up visit. The estimate of α was 0.012/month for rapid progressors (defined by λ > 0) and 0.0032/month for slow progressors (defined by λ ≤ 0) in the training group. The parameter estimates acquired from different validation folds during cross-validation procedure are shown in Table S3. Figure 2 depicts different progression patterns of rapid and slow progressors over time.

For the simulations, the maximum time was set to 300 months with 0.5 month used as the time step. To generate 50% prediction intervals, 100 stochastic simulations were performed. The simulated parameters of each patient were sampled from log-normal distributions centered at the population mean and the associated variances. For a given baseline MPG (MPG_0_), the predicted time to reach MPG of 40 mmHg ($T_{40}$) was significantly different between slow and rapid progressors. Rapid progressors yielded a much shorter $T_{40}\;$ than slow progressors at all MPG_0_. This can be observed not only in mean values, but also in 50% prediction intervals. It can be seen that $T_{40}$ values decrease with increasing MPG_0_.

Table 2 compares baseline clinical and echocardiographic characteristics between rapid and slow progressors. Total cholesterol was marginally higher in rapid progressor compared to slow progressor (*p* = 0.061), however, low density lipoprotein (LDL) cholesterol was similar between two groups. Slow progressors showed a significantly larger left ventricular (LV) end systolic dimension and lower LV ejection fraction compared to the rapid progressors, but stroke volume was similar in the two groups.

**Figure 3:**
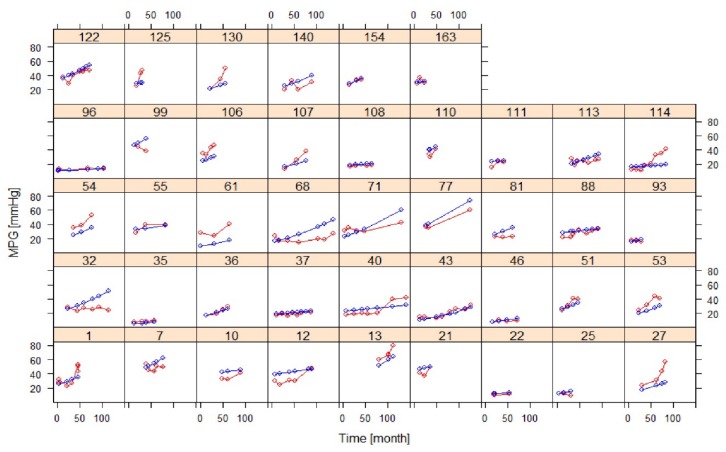
Goodness of fit plots of the AS progression model in selected individuals with at least three post-baseline measurements (training dataset) (red: observation, blue: prediction).

Figure 3 shows model fits of MPG for individuals with at least three post-baseline measurements, indicating overall good agreement between observations and predictions, except for individuals with atypical trends.

### 3.5. AD Progression Model

**Figure 4:**
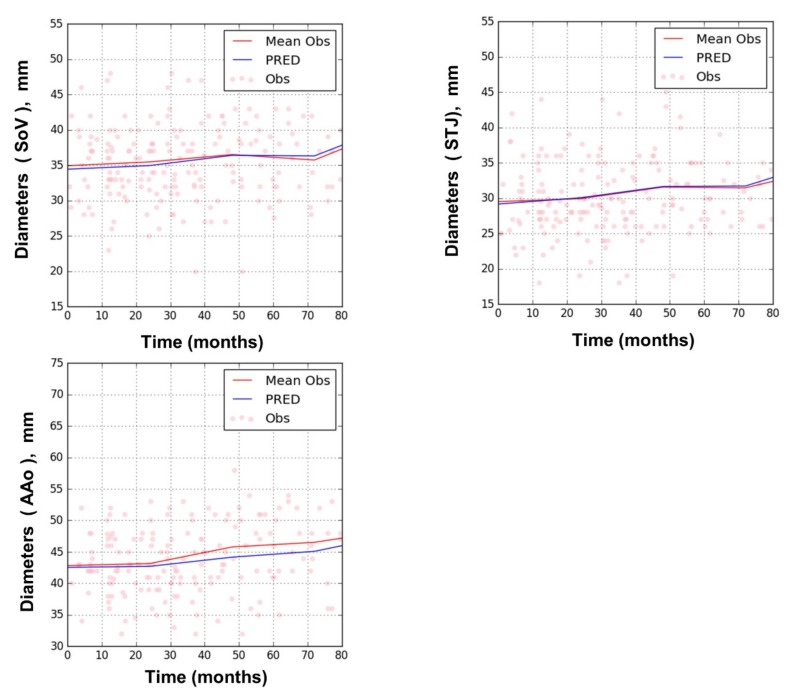
Goodness of fit plots of the AD progression model for sinus of Valsalva (SoV), sinus tubular junction (STJ), and ascending aorta (AAo); training dataset. (dots: observation, red line: mean observation, blue line: prediction).

**Figure 5:**
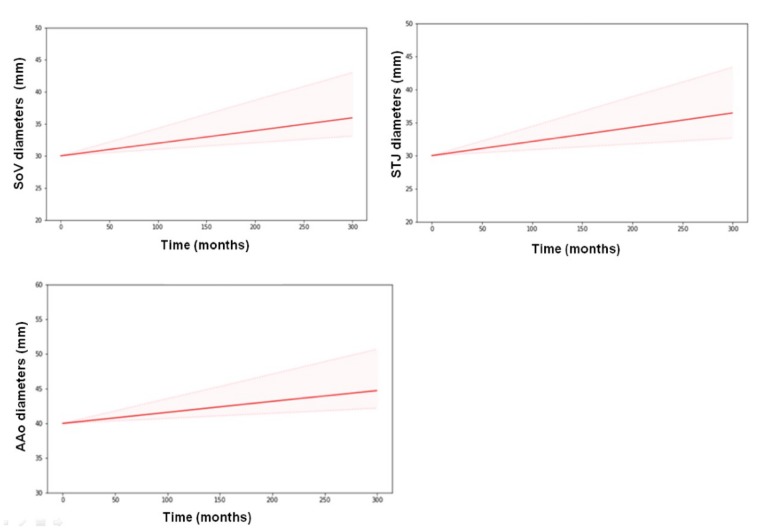
Model prediction of AD progression obtained assuming the same baseline of 30 mm for SoV and STJ and 40 mm for AAo for all individuals (training dataset). A common slope (SL) was assumed for all three variables. Thick lines represent means, and shaded areas represent 50% prediction intervals.

**Figure 6:**
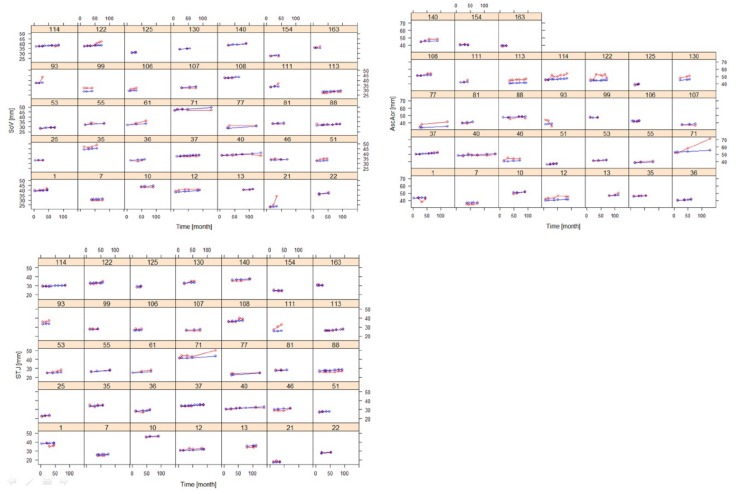
Goodness of fit plots of the AD progression model for SoV, STJ, and AAo in selected individuals with at least three post-baseline measurements (training dataset) (red: observation, blue: prediction).

The rates of increase in the diameter (SL) of SoV, STJ, and AAo did not differ significantly from each other. Hence, a common SL value was assigned to all three variables, which was estimated to be 0.019 mm/month (Table 1). No significant covariate was found to affect SL. Figure 4 shows goodness of fit plots for SoV, STJ, and AAo, indicating good agreement between observations and model predictions for all three variables. Figure 5 shows the model prediction obtained assuming the same baseline for individuals, visually representing the linear progression of AD with a rate of increment common to all three variables. Based on this model, the time to surgical threshold (T_55_) can be derived as follows: T_55_ = (55 − D_0_)/0.019. Figure 6 shows goodness of fit plots of these variables in individuals, also indicating good agreement between observations and model predictions.

### 3.6. Model Validation

Goodness of fit plots for the AS model in the validation group are presented in Figure S2 (population) and Figure S3 (selected individuals). As described in the Methods section, the predicted curves were obtained using model parameter estimates from the training group. The figure shows that AS progression in the validation group is reasonably well predicted by the model obtained using training data when first subsequent visit information is included. This further supports the validity of the method using λ to predict disease progression in patients with BAV.

The R-squared statistics of the predicted MPG were 0.76 and 0.84 in the training and validation groups, respectively. R-squared statistics of the predicted SoV, STJ, and AAo were 0.92, 0.93, and 0.82 in the training group and 0.92, 0.90, and 0.88 in the validation group, respectively. These values were significantly higher than those of the predicted MPG.

## 4. Discussion

The principal findings of the study are: (1) The disease progression of AS, as opposed to AD, is non-linear and requires a mathematical model to mimic its trajectory. A logistic time function was found to describe the overall disease progression reasonably well and enabled a rough prediction of the future time course at baseline assessment. (2) Incorporation of subsequent follow-up visit information via the covariate, λ, significantly improved the model predictability for AS compared to using baseline information only. As a result, two distinctive patterns of AS progression were identified (rapid vs. slow progressor) when MPG was analyzed, with respect to λ ≤0 and λ >0. The developed model was also able to predict the time to intervention for the rapid and slow progressor groups for AS and AD. The results obtained in this study can be used for treatment optimization in BAV patients, including risk stratification for future intervention, design of appropriate follow-up intervals, and estimation of time to future intervention for severe AS or AD.

Conventional analysis generally assumes the status of AS to be time invariant and attempts to predict disease progression by analyzing only baseline characteristics. Several prospective studies suggested risk factors for AS progression including degree of valve calcification, older age, dyslipidemia, hypercalcemia, or smoking [18,19,20,21,22,23,24]. However, considering the degenerative nature of AS, deterioration of function and morphology of the AV would accelerate over time with progression of AS. Therefore, allowing the time course of AS to be time variant would be more appropriate. In this regard, this study demonstrated a novel, model-based approach to describe the time-varying nature of disease progression of bicuspid AS and AD.

It should be noted that there were two distinctive phenotypes for AS progression determined by MPG increment rates during the subsequent follow-up visit. Baseline characteristics of slow and rapid progressors were similar, except for LV end systolic dimension and LV ejection fraction. Although the stroke volume index did not differ between the two groups, a higher LV ejection fraction in the rapid progressor group may suggest increased shear stress of the bicuspid AV. The potential importance of mechanical stress on calcific AV has been suggested by previous studies similar to our data [25]. Interestingly, there was no significant difference in lipid profiles and renal function at baseline between rapid and slow progressors. Rapid MPG increment may reflect overall dynamic influence of inflammatory or degenerative process of BAV which might not be determined by baseline information. BAV type did not differ between the rapid and slow progressor groups. Although progression rate differed according to BAV phenotype when only baseline information was considered, when subsequent follow-up data were incorporated, types of BAV lost statistical significance.

Interestingly, T_40_ was significantly shorter in rapid progressors (Figure 2B). This finding is important because current guidelines do not provide information about how fast AS can worsen or progress to the threshold for intervention. Previous large cohort studies suggested that older patients with at least moderate AS severity are likely to experience cardiovascular events, including surgical AV replacement [1,3]. However, they did not address how long it would take to reach a threshold for intervention. The practicality of our suggested AS progression model lies in its ability to predict the time to reach a surgical threshold for AS.

In our study, we suggested a bicuspid AS progression model based on MPG rather than aortic valve area (AVA) because accuracy of AVA obtained by measuring multiple echocardiographic variables can amplify modeling errors. Although AVA has been well validated in both clinical and experimental studies, MPG is easier and simpler to measure and is accompanied by a smaller measurement error [26,27]. Therefore, we derived a bicuspid AS progression model based on MPG rather than AVA.

A choice of a binary classification scheme of λ≤0 and λ>0 is geared more towards clinical utility than anything else. In fact, a more complex tripartite classification of (i) λ≤0, (ii) 0 < λ ≤0.01, and (iii) λ>0.01 has shown to lead to better predictability. Here, 0.01 was chosen, since it was the mean λ in the training dataset, indicating that the choice of 0.01 as the division boundary seems rather arbitrary since it depends on the mature of the training dataset. While searching for a better classification scheme would indeed be useful, we have decided to settle with the binary classification scheme, which is simpler and easier to implement in real clinical situations.

Our suggested AD progression model was described by linear disease progression with a constant rate of progression. According to our results, the yearly progression rate of aortic dilatation was 0.019 mm/year regardless of baseline aorta size. The progression rate of AD obtained from our study is significantly lower than the average rate reported in previous studies (1.1 ± 0.15 mm/year), even after considering the heterogeneity of age and ethnicity of the study population [27,28]. A slower progression rate in our study may have resulted from our selected study population. Detaint et al. [28] suggested that rapid progression of AD was observed in younger patients and was related to the degree of aortic regurgitation (AR). Since our study enrolled BAV patients diagnosed predominantly with AS, patients with significant AR were excluded. In addition, patients who had a significantly dilated aorta or an aortic dissection satisfying indications for surgical intervention at diagnosis were excluded from the analysis because they did not have subsequent follow-up echocardiographic data prior to the surgery.

A few limitations must be addressed in this study. First, we focused on BAV patients with predominant AS because assessment of severity is simpler compared to those with AS mixed with AR. This was also because the indication for undergoing surgical intervention for AS is more objective than in AR. Further research is required to apply our disease progression model to BAV patients with significant AR. Second, aortic diameters were measured only by transthoracic echocardiography. Although echocardiography is the first-choice imaging technique to evaluate the ascending aorta, the reproducibility for measuring segments of the aorta is limited compared to computed tomography or magnetic resonance imaging. Considering asymmetric dilatation of the ascending aorta in association with eccentric blood flow across the AV in BAV patients, measuring the diameter of the ascending aorta with echocardiography may have limitations. Further study with more reproducible imaging modality of aorta is needed to assess progression of aortopathy in BAV patients. Third, other imaging data, such as valve calcification from computed tomography, was not assessed in our study. Although calcium imaging is an alternative method which offers good prediction of disease progression, repeated use of CT is limited by cumulative radiation exposures. In addition, serial follow-up of MPG by echocardiography is currently the recommended method for monitoring hemodynamic progression of AS. Whether the distribution or burden of valve calcification would differ between rapid and slow progressor groups needs to be further studied.

## 5. Conclusions

The novel, longitudinal disease progression model described the time-varying nature of disease progression of bicuspid AS and AD, and incorporating subsequent follow-up visit data significantly improved the model predictability for AS. The proposed model can provide useful information regarding the expected time to reach a threshold for intervention. It is expected that our model will contribute to tailored management of BAV patients and facilitate risk stratification.

## Figures and Tables

**Table 1:** Parameter estimates of the final disease progression model for the training dataset.

Model Type	Parameter	Population Estimate (%RSE)
	**Structural Parameter ^§^**	
**MPG**	**α (month^−1^)**	
	$\alpha {\;}_{\lambda \;\leq \;0}$	0.0032 (27.80%)
	$\alpha_{\;\lambda \;>\;0}$	0.012 (19.00%)
	${\mathrm{MPG}}_{\infty }$ (mmHg)	95.4 (26.65%)
**SoV, STJ, and AAo**	**SL (mm/month)**	0.019 (3.62%)
	**Variance Parameter ^§§^**	
**MPG**	**α (CV%)**	72.42 (13.76%)
**SoV, STJ, and AscAor**	**SL_SoV_ (CV%)**	127.5 (9.29%)
	**SL_STJ_ (CV%)**	90.4 (12.4%)
	**SL_AscAor_**	116.6 (8.38%)

**^§^** In the model of SoV, STJ, and AAo, SL was assumed to be the same for all three variables. **^§§^** In the variance parameter, CV denotes coefficient of variation. %RSE: percent relative standard error, MPG: mean pressure gradient; SoV, sinus of Valsalva; STJ, sinus tubular junction; AAo, ascending aorta; SL, slope.

**Table 2:** Comparison of patient demographics and covariates between rapid and slow progressors.

Characteristics	Rapid Progressor (*n* = 80)	Slow Progressor (*n* = 46)	*p*-Value
**Clinical characteristics**			
Age, year	61 (27–86)	63 (27–82)	0.947
Male gender, (%)	47 (59)	27 (59)	1.000
Body weight, kg	65.3 ± 16.5	62.4 ± 10.2	0.225
Systolic BP, mmHg	123.6 ± 17.3	119.4 ± 17.8	0.195
Diastolic BP, mmHg	77.4 ± 11.8	74.0 ± 13.4	0.150
Hypertension	28 (48)	4 (50)	0.999
Diabetic mellitus	12 (21)	0 (0)	0.333
Dyslipidemia	20 (35)	2 (25)	0.709
Atrial fibrillation	5 (9)	1 (13)	0.555
Prior myocardial infarction	1 (2)	1(13)	0.229
Previous history of stroke	3 (5)	0 (0)	0.999
ACEi/ARB	32 (55)	3 (38)	0.459
Statin	20 (35)	2 (25)	0.709
Calcium channel blockers	11 (19)	2 (25)	0.651
Beta blockers	23 (40)	3 (38)	0.999
Hemoglobin	13.6 ± 2.0	13.4 ± 1.6	0.780
Log NT-proBNP	2.25 ± 0.80	3.28 ± 0.60	0.338
Total cholesterol	179.0 ± 29.7	154.7 ± 29.3	0.061
LDL-cholesterol	104.8 ± 28.1	98.2 ± 20.5	0.578
eGFR	77.8 ± 21.8	78.6 ± 19.0	0.932
**Echocardiographic characteristics**			
LV EDD, mm	51.2 ± 6.9	52.8 ± 9.2	0.292
LV ESD, mm	33.8 ± 6.9	37.5 ± 10.1	0.032
LV mass index, mg/m^2^	124.1 ± 46.9	136.0 ± 59.5	0.247
LV ejection fraction, %	67 ± 7	57 ± 17	<0.001
LA volume index, ml/	29.6 ± 15.8	33.2 ± 18.7	0.270
Stroke volume, ml	84.5 ± 21.0	84.1 ± 36.8	0.950
MPG, mmHg	27.3 ± 16.4	29.5 ± 19.3	0.509
AVA, cm^2^	1.22 ± 0.38	1.21 ± 0.38	0.978
SoV, mm	35.0 ± 5.2	33.6 ± 5.6	0.155
STJ, mm	29.8 ± 5.3	28.9 ± 5.8	0.412
AAo, mm	41.7 ± 5.5	42.4 ± 4.9	0.469
**BAV phenotypes**			
Type I	43 (54)	23 (50)	0.491
Type II	10 (130)	8 (17)	
Type III	7 (9)	1 (2)	
Type IV	19 (24)	14 (30)	
Undetermined	1 (1)	0 (0)	

Values were expressed as mean ± SD, median (range) or number (%); BP, blood pressure; ACEi, Angiotensin converting enzyme inhibitors; ARB, angiotensin receptor blockers; LDL, low density lipoprotein; eGFR, estimated glomerular filtration rate; LV, left ventricle; EDD, end diastolic dimension; ESD, end systolic dimension; LA, left atrium; MPG, mean pressure gradient; AVA, aortic valve area; BAV, bicuspid aortic valve; NT-proBNP, N-terminal pro b-type Natriuretic Peptide.

## Supplementary Materials

The following are available online at https://www.mdpi.com/2077-0383/8/9/1302/s1, Figure S1: Linear regression of MPG vs. time in selected patients, Figure S2: Goodness of fit plots of the AS progression model stratified by λ, Figure S3: Goodness of fit plots of the AS progression model in selected individuals with at least three post-baseline measurements, Table S1: Clinical and echocardiographic parameters of patients in the training and validation groups, Table S2: Four-fold cross-validation results, Table S3: The parameter estimates of the selected logistic disease progression model in the different folds, Table S4: Mean estimated slope coefficients from linear regression analyses of MPG on time stratified by baseline MPG groups.
