# Supplementary material for: Predicting Disease Progression in Patients with Bicuspid Aortic Stenosis Using Mathematical Modeling
Source: J Clin Med. 2019 Aug 24;8(9):1302. doi: 10.3390/jcm8091302 (PMC6780906; doi:10.3390/jcm8091302)
Supplement: Supplementary file 1 [file jcm-08-01302-s001.pdf]

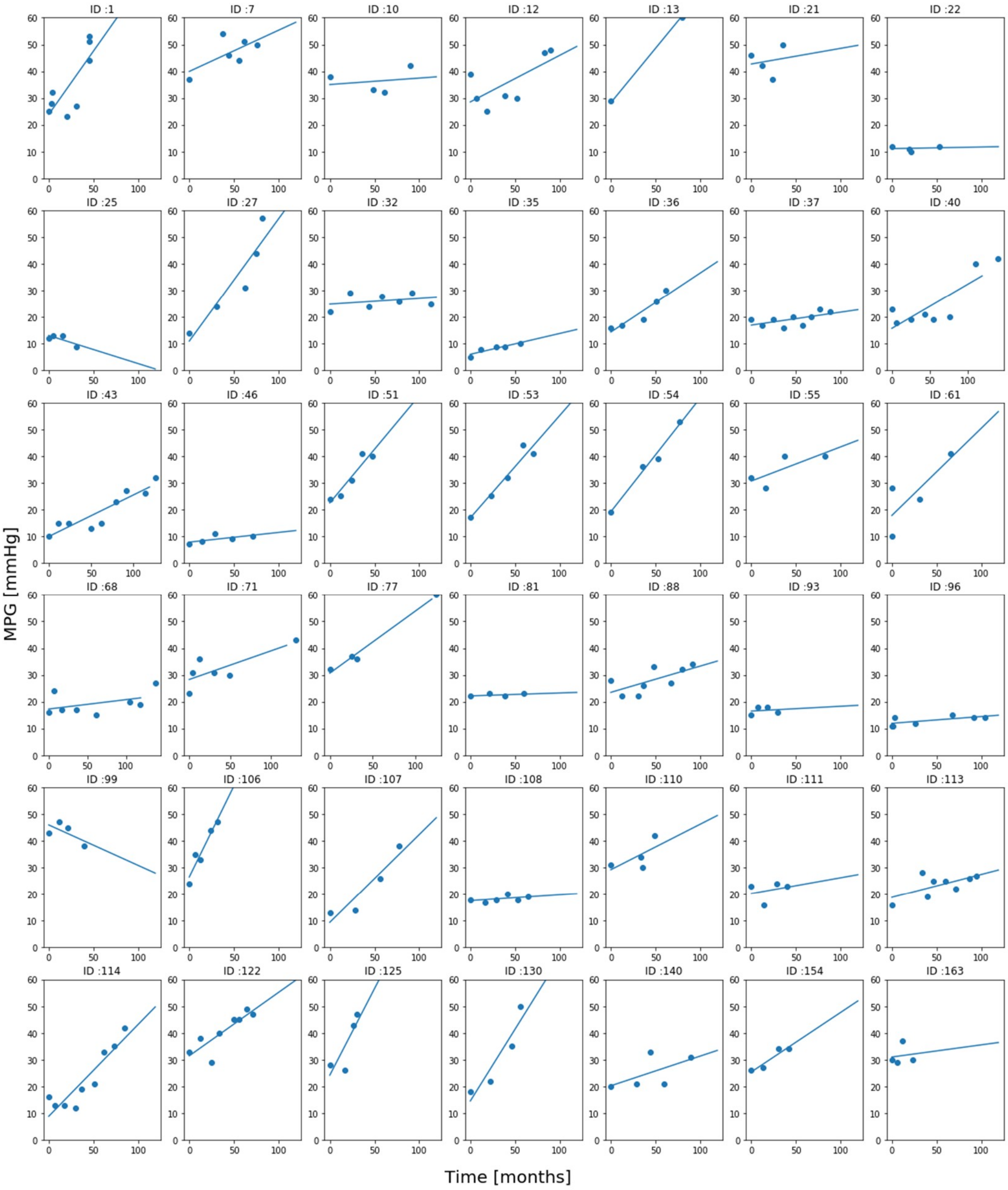

Figure S1. Linear regression of MPG vs. time in selected patients.

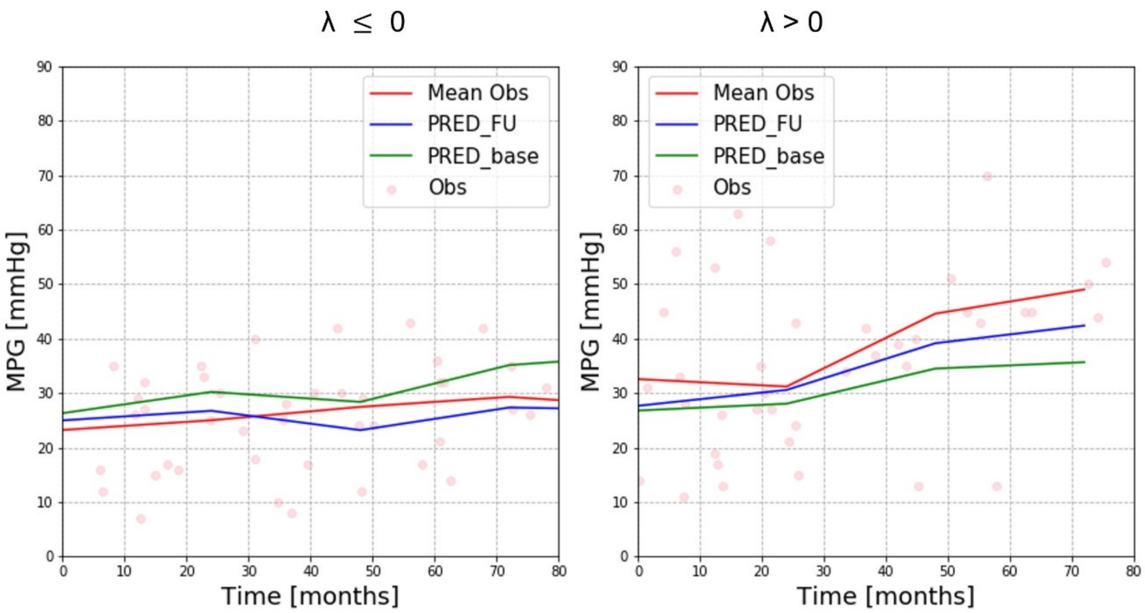

Figure S2. Goodness of fit plots of the AS progression model stratified by λ (validation dataset) (dots: observation, red line: mean observation, blue line: mean prediction with incorporation of subsequent visit information, green line: mean prediction with only baseline information)

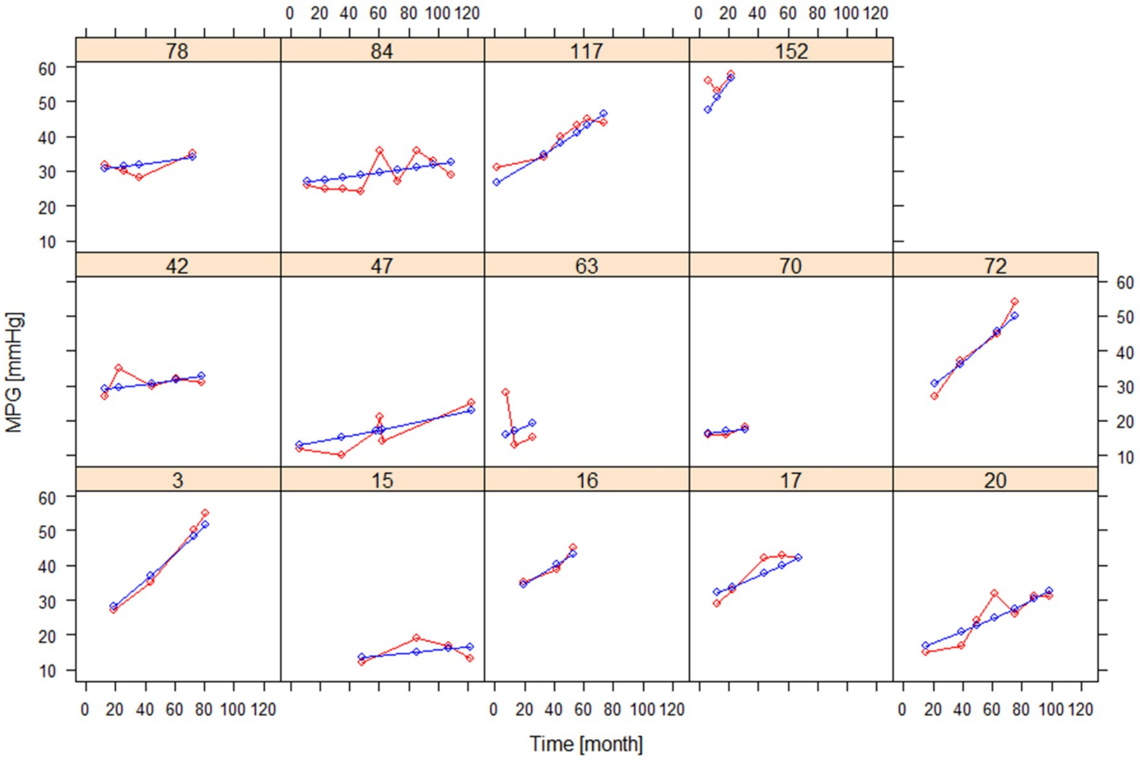

Figure S3. Goodness of fit plots of the AD progression model in selected individuals with at least three post-baseline measurements (validation dataset) (red: observation, blue: prediction).

Table S1. Clinical and echocardiographic parameters of patients in the training and validation groups

Characteristics	Training Group (n = 126)	Validation Group (n = 43)	p-value
**Clinical characteristics**			
Age, year (median, range)	61(27–86)	59 (35–78)	0.842
Male gender, (%)	74 (59)	31 (72)	0.173
Body weight, kg	64.3 ± 14.6	66.9 ± 10.4	0.204
Body surface area, m^2	1.69 ± 0.18	1.74 ± 0.14	0.102
Systolic BP, mmHg	122.1 ± 17.5	121.6 ± 15.2	0.863
Diastolic BP, mmHg	76.2 ± 12.5	75.53 ± 9.8	0.742
Hypertension	63 (50)	18 (42)	0.228
Diabetic mellitus	25 (20)	8 (19)	0.999
Dyslipidemia	43 (34)	16 (37)	0.715
Atrial fibrillation	13 (10)	5 (12)	0.780
Prior myocardial infarction	3 (2)	1 (2)	1.000
Previous history of stroke	4 (3)	1 (2)	0.999
ACEi/ARB	62 (50)	22 (51)	0.861
Statin	43 (34)	16 (37)	0.715
Calcium channel blockers	27 (21)	10 (23)	0.832
Beta blockers	44 (35)	14 (33)	0.854
Hemoglobin	14.0 ± 4.5	13.8 ± 1.9	0.771
Log NT-proBNP	2.93 ± 1.10	2.67 ± 0.88	0.630
Total cholesterol	175.8 ± 34.3	175.1 ± 37.3	0.903
LDL-cholesterol	105.8 ± 34.8	101.7 ± 33.1	0.534
eGFR	78.6 ± 22.0	86.4 ± 17.7	0.039
**Echocardiographic characteristics**			
LV EDD, mm	51.7 ± 7.8	49.9 ± 7.8	0.182
LV ESD, mm	35.2 ± 8.3	34.3 ± 7.3	0.253
LV mass index, mg/m^2	128.5 ± 52.2	117.5 ± 55.6	0.242
LV ejection fraction, %	66 ± 9	67 ± 8	0.431
LA volume index, ml/	30.9 ± 16.9	27.9 ± 12.5	0.283
Stroke volume, ml/ m^2	83.3 ± 29.8	81.7 ± 20.3	0.762
MPG, mmHg	27.9 ± 17.1	26.4 ± 17.3	0.623
AV VTI, cm			
AVA, cm^2	1.22 ± 0.39	1.27 ± 0.43	0.525
SoV, mm	34.5 ±5.3	35.9 ± 6.0	0.139
STJ, mm	29.4 ± 5.5	30.6 ± 7.0	0.287
AAo, mm	42.0 ± 5.9	41.0 ± 7.9	0.451
BAV phenotypes			0.452
Type I	66 (53)	23 (53)	
Type II	18 (14)	5 (12)	
Type III	8 (6)	6 (14)	

Type IV	33 (26)	9 (21)	
Unknown	1 (1)	0 (0)	
**No. of measurements per patient**	3.0 (2.0–9.0)	3.0 (2.0–10.0)	0.801

Mean ± SD, (median, range); BP, blood pressure; ACEi, Angiotensin converting enzyme inhibitors; ARB, angiotensin receptor blockers; eGFR, estimated glomerular filtration rate; LV, left ventricle; EDD, end diastolic dimension; ESD, end systolic dimension; LA, left atrium; MPG, mean pressure gradient; AV, aortic valve; VTI, velocity time integral; AVA, aortic valve area; BAV, bicuspid aortic valve.

Table S2. Four-fold cross-validation results

Training/Test Folds	OFV	Linear	Asymptotic Expo	Logistic
2,3,4/1	Training folds	1321.15	1321.15	1307.36
	Validation fold	628.75	628.13	624.57
1,3,4/2	Training folds	1462.72	1460.51	1445.85
	Validation fold	456.85	456.57	456.33
1,2,4/3	Training folds	1440.18	1434.54	1430.32
	Validation fold	480.46	480.81	472.59
1,2,3/4	Training folds	1518.58	1512.26	1501.51
	Validation fold	401.67	400.83	400.14
Sum of OFV	-	1967.73	1966.34	1953.63

Table S3. The parameter estimates of the selected logistic disease progression model in the different folds

Training/Test Folds	Model Parameters
2,3,4/1	α ($\lambda \leq 0$): 0.0040/month<br>α ($\lambda > 0$): 0.013/month<br>MPG_{∞}: 81.8 mmHg
1,3,4/2	α ($\lambda \leq 0$): 0.0034/month<br>α ($\lambda > 0$): 0.013/month<br>MPG_{∞}: 90.3 mmHg
1,2,4/3	α ($\lambda \leq 0$): 0.0031<br>α ($\lambda > 0$): 0.011<br>MPG_{∞}: 104.0 mmHg
1,2,3/4	α ($\lambda \leq 0$): 0.0031/month<br>α ($\lambda > 0$): 0.012/month<br>MPG_{∞}: 83.3 mmHg

Table S4. Mean estimated slope coefficients from linear regression analyses of MPG on time stratified by baseline MPG groups

Baseline MPG	Estimated slope coefficients
5–10 mmHg	0.17 mmHg/month
10–20 mmHg	0.18 mmHg/month
20–30 mmHg	0.26 mmHg/month
30–40 mmHg	0.16 mmHg/month
40–50 mmHg	−0.046 mmHg/month
